# Membrane-Sculpting BAR Domains Generate Stable Lipid Microdomains

**DOI:** 10.1016/j.celrep.2013.08.024

**Published:** 2013-09-19

**Authors:** Hongxia Zhao, Alphée Michelot, Essi V. Koskela, Vadym Tkach, Dimitrios Stamou, David G. Drubin, Pekka Lappalainen

**Affiliations:** 1 Institute of Biotechnology, University of Helsinki, 00014 Helsinki, Finland; 2 Department of Molecular and Cell Biology, University of California, Berkeley, CA 94720-3202, USA; 3 Bio-Nanotechnology Laboratory, Department of Chemistry and Nano-Science Center, University of Copenhagen, 2100 Copenhagen, Denmark; 4 Lundbeck Foundation Center Biomembranes in Nanomedicine, University of Copenhagen, 2100 Copenhagen, Denmark

## Abstract

Bin-Amphiphysin-Rvs (BAR) domain proteins are central regulators of many cellular processes involving membrane dynamics. BAR domains sculpt phosphoinositide-rich membranes to generate membrane protrusions or invaginations. Here, we report that, in addition to regulating membrane geometry, BAR domains can generate extremely stable lipid microdomains by “freezing” phosphoinositide dynamics. This is a general feature of BAR domains, because the yeast endocytic BAR and Fes/CIP4 homology BAR (F-BAR) domains, the inverse BAR domain of Pinkbar, and the eisosomal BAR protein Lsp1 induced phosphoinositide clustering and halted lipid diffusion, despite differences in mechanisms of membrane interactions. Lsp1 displays comparable low diffusion rates in vitro and in vivo, suggesting that BAR domain proteins also generate stable phosphoinositide microdomains in cells. These results uncover a conserved role for BAR superfamily proteins in regulating lipid dynamics within membranes. Stable microdomains induced by BAR domain scaffolds and specific lipids can generate phase boundaries and diffusion barriers, which may have profound impacts on diverse cellular processes.

## INTRODUCTION

Cellular processes, such as endocytosis, cell migration, and morphogenesis, require precise regulation of plasma membrane shape and dynamics. In addition to actin polymerization, which produces forces for generation of plasma membrane protrusions and invaginations, these processes rely on proteins that bind directly to membranes to sense membrane curvature and sculpt them into desired shapes (Graham and Kozlov, 2010; McMahon and Gallop, 2005; Antonny, 2011). Among the central membrane-sculpting proteins are the members of the Bin-Amphiphysin-Rvs (BAR) domain superfamily. The BAR domain is a dimeric a-helical protein motif, which interacts with membranes through a curved interface. Furthermore, the BAR domains can oligomerize into helical scaffolds to further promote membrane deformation (Frost et al., 2008; Mim et al., 2012; Peter et al., 2004; Shimada et al., 2007; Takei et al., 1999). Depending on the geometry of the lipid-binding interface and oligomerization properties of the domain, BAR superfamily domains can generate either positive (BAR and most Fes/CIP4 homology BAR [F-BAR] domains) or negative membrane curvature (most inverse BAR [I-BAR] domains) as well as stabilize planar membrane sheets (the I-BAR domain of Pinkbar; Frost et al., 2009; Pykäläinen et al., 2011; Suetsugu et al., 2010; Zhao et al., 2011). In addition to electrostatic interactions with lipid head groups, a subset of canonical BAR domains (N-terminal BAR [N-BAR] domains) and certain I-BAR domains can insert amphipathic a helices into the lipid bilayer. In N-BAR domain-induced membrane tubules, the N-terminal amphipathic α helices provide important interdomain contacts to stabilize the N-BAR scaffolds (Mim et al., 2012). The membrane-inserting α helices of BAR domains have also been reported to drive membrane scission, regulate the diameter of membrane tubules, and sense positive membrane curvature (Bhatia et al., 2009; Boucrot et al., 2012; Gallop et al., 2006; Masuda et al., 2006; Saarikangas et al., 2009).

Formation of plasma membrane invaginations during endocytosis is a complex process that requires both the actin polymerization machinery and generation of positive membrane curvature by BAR and F-BAR domain proteins (Collins et al., 2011; Kukulski et al., 2012; Qualmann et al., 2011). In budding yeast, two F-BAR domain proteins, Syp1 and Bzz1, and a heterodimeric BAR domain protein, Rvs161/167, contribute to endocytosis. These three membrane-sculpting proteins are recruited to the endocytic sites with distinct timing. Syp1 is among the first proteins to arrive, but it departs from the endocytic site before formation of a deep membrane invagination and scission, which coincide with the arrival of Bzz1 and Rvs161/167 at the site (Weinberg and Drubin, 2012). Bzz1, acting at the invagination base, stabilizes endocytic sites and functions with Rvs161/167, localized along the tubule, to achieve proper endocytic membrane geometry necessary for efficient scission (Kishimoto et al., 2011). However, the biochemical differences between various BAR and F-BAR domains that may underlie their sequential recruitment and specific functions in endocytic patches are not known.

A recent study revealed that mammalian I-BAR domains and the BAR domain of amphiphysin can induce the clustering of phosphatidylinositol 4,5-bisphosphate (PI(4,5)P_2_) (Saarikangas et al., 2009). Furthermore, theoretical work proposed that membrane-scission during endocytosis in budding yeast may result from a lipid-phase boundary induced by PI(4,5)P_2_ clustering via BAR domains at the neck of endocytic invagination (Liu et al., 2009). However, whether the BAR domain scaffolds can limit the lateral mobility of lipids in membranes to generate stable lipid-phase boundaries and diffusion barriers has not been reported. Here, we revealed that BAR domains assemble into stable scaffolds, which not only bend membranes but also inhibit the lateral diffusion of phosphoinositide molecules by at least two orders of magnitude. Generation of extremely stable protein-lipid microdomains appears to be a general feature of BAR superfamily proteins, because all membrane-tubulating BAR and F-BAR domains tested here, as well as the BAR domain of Pinkbar, which stabilizes planar membrane sheets, efficiently halted phosphoinositide diffusion. Our results suggest that BAR domain scaffolds form lipid diffusion barriers and phase boundaries, which are likely to have profound importance in a wide variety of cellular processes.

## RESULTS

### Yeast Endocytic BAR and F-BAR Domains Are Biochemically Distinct

To examine the general biochemical properties of the BAR/F-BAR domains of budding yeast endocytic proteins Syp1, Bzz1, and Rvs161/167 and to reveal their possible differences, these domains were produced as nontagged versions and as GFP/ mCherry-fusion proteins in *E. coli*. As reported previously, the purified F-BAR domain of Syp1 and BAR domain of Rvs161/ 167 (Reider et al., 2009; Youn et al., 2010), as well as the previously uncharacterized F-BAR domain of Bzz1, induced tubulation of PI(4,5)P_2_-containing vesicles. Based on electron microscopy analysis, the diameters of membrane tubules generated by the three domains were similar to each other (20 nm ± 2.5 nm; Figure S1A). However, time-lapse imaging of giant unilamellar vesicles (GUVs) in the presence of the protein domains suggested that the heterodimeric Rvs161/167 BAR domain is slightly more efficient in tubulating membranes compared to the two endocytic F-BAR domains (Figures S1B and S1C). Furthermore, slightly higher concentration of Syp1 F-BAR domain (~1 µM) was required for efficient membrane tubulation compared to Rvs161/167 BAR domain and Bzz1 F-BAR domain (~0.5 µM) (data not shown).

**Figure 1:**
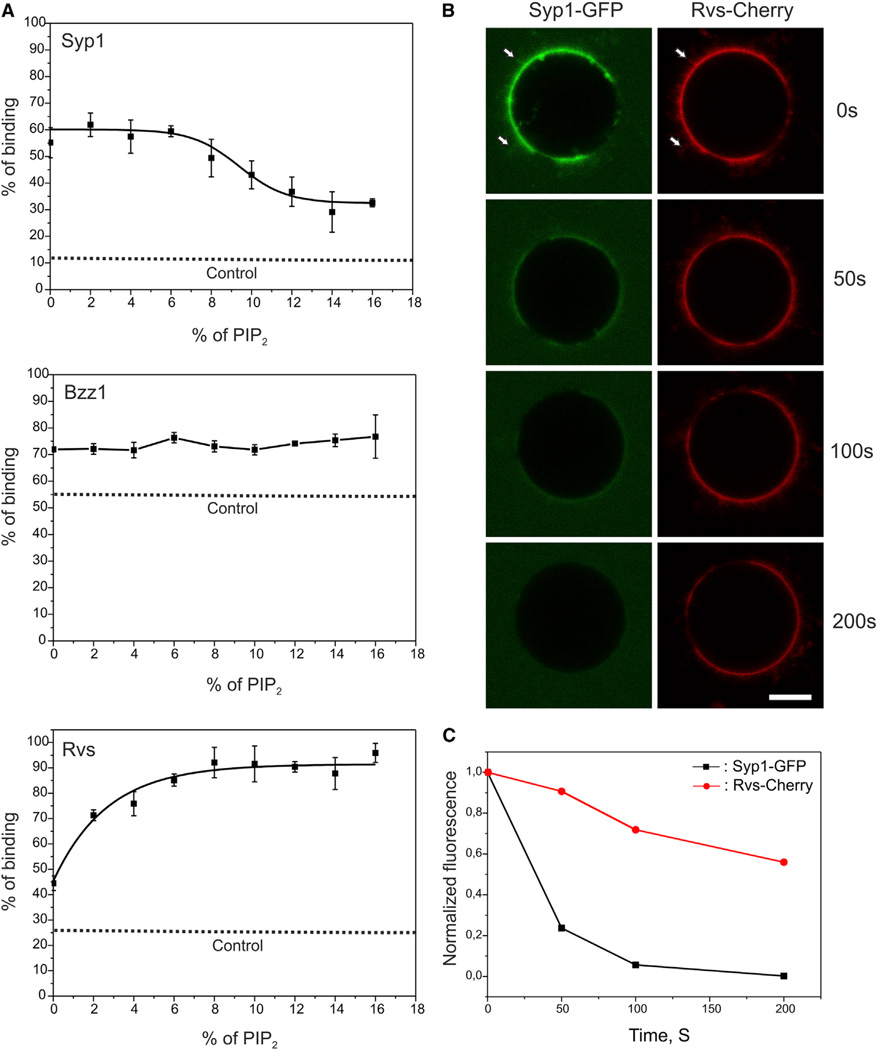
Yeast Endocytic BAR/F-BAR Domains Display Differences in Their Lipid Specificities (A) The F-BAR/BAR domains of Syp1, Bzz1, and Rvs161/167 display different PI(4,5)P_2_ specificities, as measured by a vesicle cosedimentation assay. The membrane binding of Syp1 F-BAR domain was slightly inhibited at high (>8%) PI(4,5)P_2_ density. In contrast, interaction of the BAR domain of Rvs161/167 with vesicles was significantly enhanced by PI(4,5)P_2_, whereas the membrane binding of Bzz1 F-BAR domains was not augmented by high PI(4,5)P_2_ density in the membranes. The data are from three independent experiments, and the error bar indicates ± SD. The dotted line (control) indicates the amount of protein sedimenting in the absence of lipids. (B) When added simultaneously on vesicles, the BAR/F-BAR domains of Rvs161/167 (Rvs-Cherry) and Syp1 (Syp1-GFP) initially bind to GUVs and colocalize to PI(4,5)P_2_-rich tubules (indicated by arrows). However, the Syp1 F-BAR domain dissociates from the membrane within 1 to 2 min, whereas the BAR domain of Rvs 161/167 remains bound to the surface of GUVs. The scale bar represents 10 µM. (C) Quantification of the relative fluorescence intensities of the two domains on GUVs demonstrates that Rvs161/167 BAR domain replaces the Syp1 F-BAR domain from the membrane. Please note that the slight decrease in the Rvs161/167 mCherry fluorescence most likely results from photobleaching during the monitoring period. The lipid composition was POPC:POPE:POPS:PI(4,5)P_2_ = 50:20:20:10. The concentration of the proteins was 1 µM.

Interestingly, these domains displayed prominent differences in their lipid specificities (Figures 1A and S2). Based on vesicle cosedimentation assays, interaction of the Rvs161/167 BAR domain with vesicles was strongly enhanced by PI(4,5)P_2_, whereas the membrane-binding of Bzz1 F-BAR domain was not augmented by high PI(4,5)P_2_ density of the membranes, and the membrane binding of the Syp1 F-BAR domain was slightly inhibited at high (>8%) PI(4,5)P_2_ density (Figure 1A). Because a large fraction of Bzz1 F-BAR domain sedimented, even in the absence of vesicles, its lipid specificity was also examined by tryptophan fluorescence assay, which provided very similar results to the cosedimentation assay (Figure S2D). Possible specificity of the Rvs161/167 BAR domain toward certain phosphoinositides was further examined by a vesicle cosedimentation assay. The data revealed that this domain binds to vesicles containing 10% phosphoinositides with an affinity of PI(3,4,5)P_3_ > PI(4,5)P_2_ > PI3P (Figures S2E and S2F).

Consistent with the biochemical data, time-lapse imaging of GUVs incubated simultaneously with Syp1 F-BAR and Rvs161/ 167 BAR domains revealed that, although the two domains can initially colocalize on membrane microdomains and tubules (arrows in Figure 1B), the Rvs161/167 BAR domain efficiently replaces the Syp1 F-BAR domain from the surface of the PI(4,5)P_2_-containing vesicles within 1 to 2 min (Figures 1B and 1C). Furthermore, a vesicle cosedimentation experiment demonstrated that the Rvs161/167 BAR domain and Syp1 F-BAR domain compete with each other for membrane binding (Figure S1D). Therefore, similar to the situation at the endocytic sites, Rvs161/167 can replace Syp1 on membrane tubules.

In addition to their distinct lipid specificities, the three domains displayed different effects on membrane properties, including membrane fluidity and phospholipid order as detected by diphenylhexatriene (DPH) anisotropy and Laurdan generalized polarization, respectively (Parasassi et al., 1990; Zaritsky et al., 1985). The BAR domain of Rvs161/167 increased the DPH anisotropy, suggesting that it may interact with the acylchain region of the lipid bilayer (Figure S3A). In addition, Laurdan generalized polarization was increased, indicating augmented lipid packing in the interfacial region of membranes (Figure S3C), which can be caused by both protein insertion and PI(4,5)P_2_ clustering. Interestingly, these effects were diminished when the N-terminal helix of the BAR domain was deleted or the amphipathic nature of the helix was disrupted (Figures S3B and S3D; data not shown), suggesting that the N-terminal helix is important for causing the changes of these membrane phys-iochemical properties. Furthermore, mutations in the N-terminal helix diminished the affinity of the Rvs161/167 BAR domain to phosphoinositide-rich membrane by ~10-fold (Figure S2F). Importantly, changes of tryptophan (Trp3 of Rvs161) spectra (Figure S4A) and quenching of Trp3 by lipids brominated along the acylchains (Figure S4B) provided strong evidence that the N-terminal *a* helix of Rvs161 indeed inserts into the hydrophobic core region of the lipid bilayer. The effects of the Rvs161/167 BAR domain on membrane fluidity and lipid order are dependent on lipid composition. The BAR domain of Rvs161/167 affect the membrane fluidity and lipid order only in the presence of PI(4,5)P_2_ in the membrane (Figures S3E and S3F), further indicating that proper membrane interaction of the Rvs161/167 BAR domain is dependent on the phosphoinositide.

In contrast, the F-BAR domains of Syp1 and Bzz1 did not have detectable effects on the acylchain region as measured by changes in DPH anisotropy and Laurdan generalized polarization (Figures S3A and S3C). However, the F-BAR domains of Syp1 and Bzz1 displayed strong membrane curvature-sensing activity (Figure S5). These results are in apparent contradiction with previous measurements, concluding that hydrophobic insertions are essential for sensing of membrane curvature in other BAR domains (Bhatia et al., 2009). Thus, further studies are required to reveal the exact mechanism by which the F-BAR domains of Syp1 and Bzz1 sense membrane curvature. One possible explanation could be “protein crowding”, which was recently shown to induce membrane curvature (Stachowiak et al., 2012) and may also assist in curvature sensing. Alternatively, the F-BAR domains of Syp1 and Bzz1 may display very shallow insertion into the lipid bilayer, which would not be detected by DPH anisotropy and Laurdan generalized polarization methods.

Together, these data demonstrate that the BAR and F-BAR domains of the three budding yeast endocytic proteins display significant differences in their lipid specificities and in mechanisms of membrane interactions. These biochemical differences may at least partially account for the specific roles and recruitment timing of these proteins in endocytosis.

### Phosphoinositide Clustering Is a General Feature of BAR Superfamily Domains

**Figure 2:**
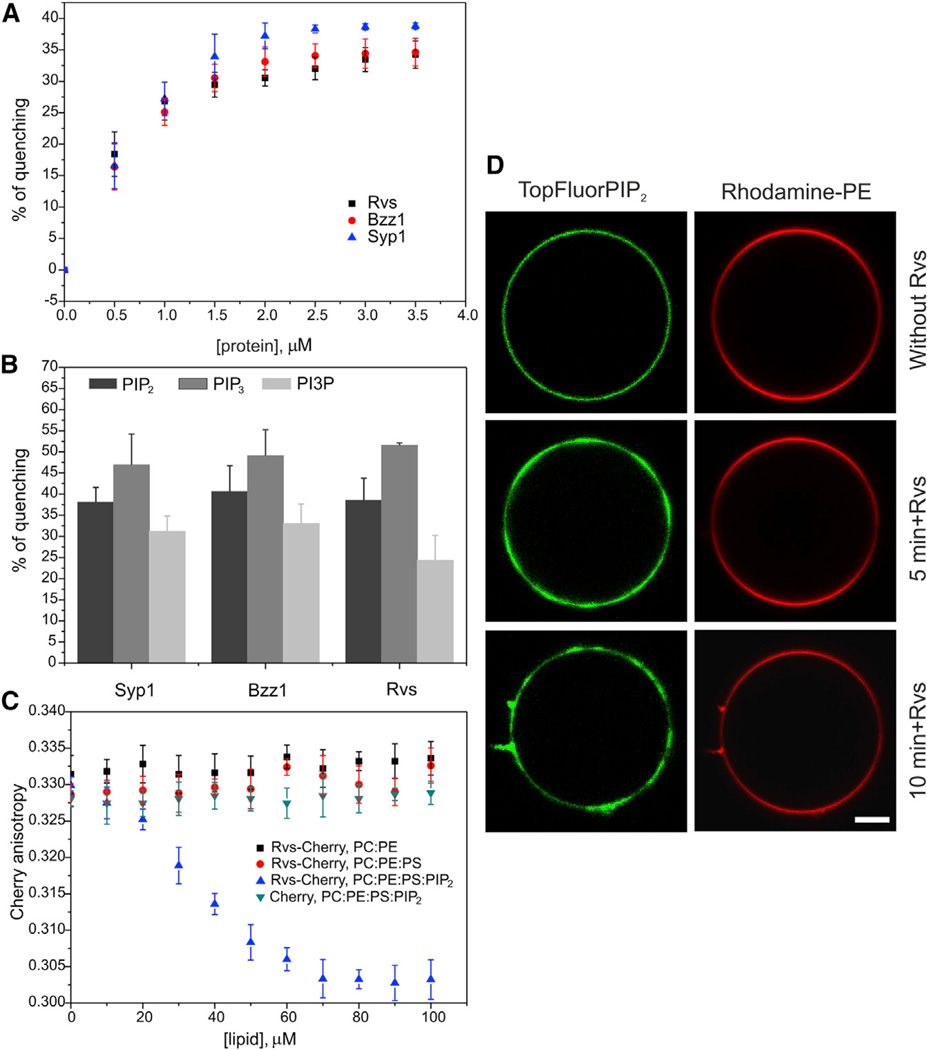
Yeast Endocytic BAR/F-BAR Domains Promote PI(4,5)P_2_ Clustering and Induce the Formation of Lipid Microdomains (A) PI(4,5)P_2_ clustering examined at the nanometer scale by measuring the quenching of BODIPY-TMR-PI(4,5)P_2_. The F-BAR/BAR domains of Syp1, Bzz1, and Rvs161/167 promoted the quenching of BODIPY-conjugated PI(4,5)P_2_ in a concentration-dependent manner, indicating that they induce phosphoinositide clustering by bringing BODIPY-TMR-PI(4,5)P_2_ molecules in close proximity to each other. The lipid composition was POPC: POPE:POPS:PI(4,5)P_2_:bodipy-TMR-PI(4,5)P_2_ = 50:20:20:9.5:0.5. All error bars indicate ± SD. (B) The F-BAR/BAR domains of Syp1, Bzz1, and Rvs161/167 clustered PI(4,5)P_2_, PI(3,4,5)P_3_, and PI3P with an efficiency of PI(3,4,5)P_3_ > PI(4,5)P_2_ > PI3P. The lipid composition was POPC:POPE: POPS:BODIPY-phosphoinositide = 59:20:20:1, and the lipid concentration was 40 µM. (C) The steady-state homo-FRET fluorescence anisotropy of Rvs-Cherry decreased in the presence of PI(4,5)P_2_-containing vesicles, suggesting that Rvs161/167 BAR domain self-assembled into oligomers in the presence of PI(4,5)P_2_. The concentration of Rvs-Cherry was 0.5 µM. (D) Formation of lipid microdomains at micrometer scale revealed by light microscopy imaging of GUVs. The F-BAR/BAR domains of Syp1, Bzz1, and Rvs161/167 generated visible TopFluor-PI(4,5)P_2_ clusters on GUVs. However, the zwitterionic lipid phosphatidylethanolamine (PE) remained mostly uniformly distributed on the GUV membrane with some PE clustering to the membrane tubules. The lipid composition was POPC:POPE:POPS:PI(4,5)P_2_:TopFluor-PI(4,5)P_2_ :Rhodamine-PE = 50:19:20:9:1: 1. The final concentration of the Rvs161/167 BAR domain was 1 µM. All experiments were carried out at room temperature. The scale bar represents 10 µM.

Mammalian I-BAR domains and the BAR domain of amphiphysin induce phosphoinositide clustering (Saarikangas et al., 2009). To determine whether this is a general activity of all BAR superfamily domains, we examined the PI(4,5)P_2_-clustering activities of Syp1, Bzz1, and Rvs161/167 BAR/F-BAR domains by a fluorometric assay and by time-lapse imaging of fluorescently labeled lipids on GUVs. The F-BAR/BAR domains of Syp1, Bzz1, and Rvs161/167 promoted quenching of BODIPY-conjugated PI(4,5)P_2_ in a concentration-dependent manner, indicating that these domains induce phosphoinositide clustering at a nanometer scale (Figure 2A). Besides PI(4,5)P_2_, the F-BAR/BAR domains of Syp1, Bzz1, and Rvs161/167 also cluster PI(3,4,5) P_3_ and PI3P with an efficiency of PI(3,4,5)P_3_ > PI(4,5)P_2_ > PI3P (Figure 2B), suggesting that the phosphoinositide clustering is mainly mediated through electrostatic interactions.

Homo-fluorescence resonance energy transfer (FRET) anisotropy experiments on the Rvs161/167 BAR domain suggested that individual BAR domain heterodimers assemble into oligomers on phosphoinositide-rich membranes (Figure 2C), as previously shown for mammalian BAR and F-BAR domains by cryoelectron microscopy (Frost et al., 2008; Mim et al., 2012). Therefore, this domain may also induce formation of larger phosphoinositide clusters through self-assembly. In support of this possibility, PI(4,5)P_2_ clustering by the F-BAR/BAR domains of Syp1, Bzz1, and Rvs161/167 was also detected by fluorescence microscopy (Figure 2D). When the Rvs161/167 BAR domain was added to GUVs containing fluorescently labeled lipids, the zwitterionic lipid phosphatidylethanolamine (PE) remained mostly uniformly localized around the GUV membrane, whereas PI(4,5)P_2_ formed visible clusters (Figure 2D). Together, these data show that the BAR and F-BAR domains of yeast endocytic proteins induce PI(4,5)P_2_ clustering, thus suggesting that phosphoinositide clustering is a general property of all BAR superfamily domains.

### Generation of Extremely Stable PI(4,5)P_2_ Microdomains by BAR Domains

**Figure 3:**
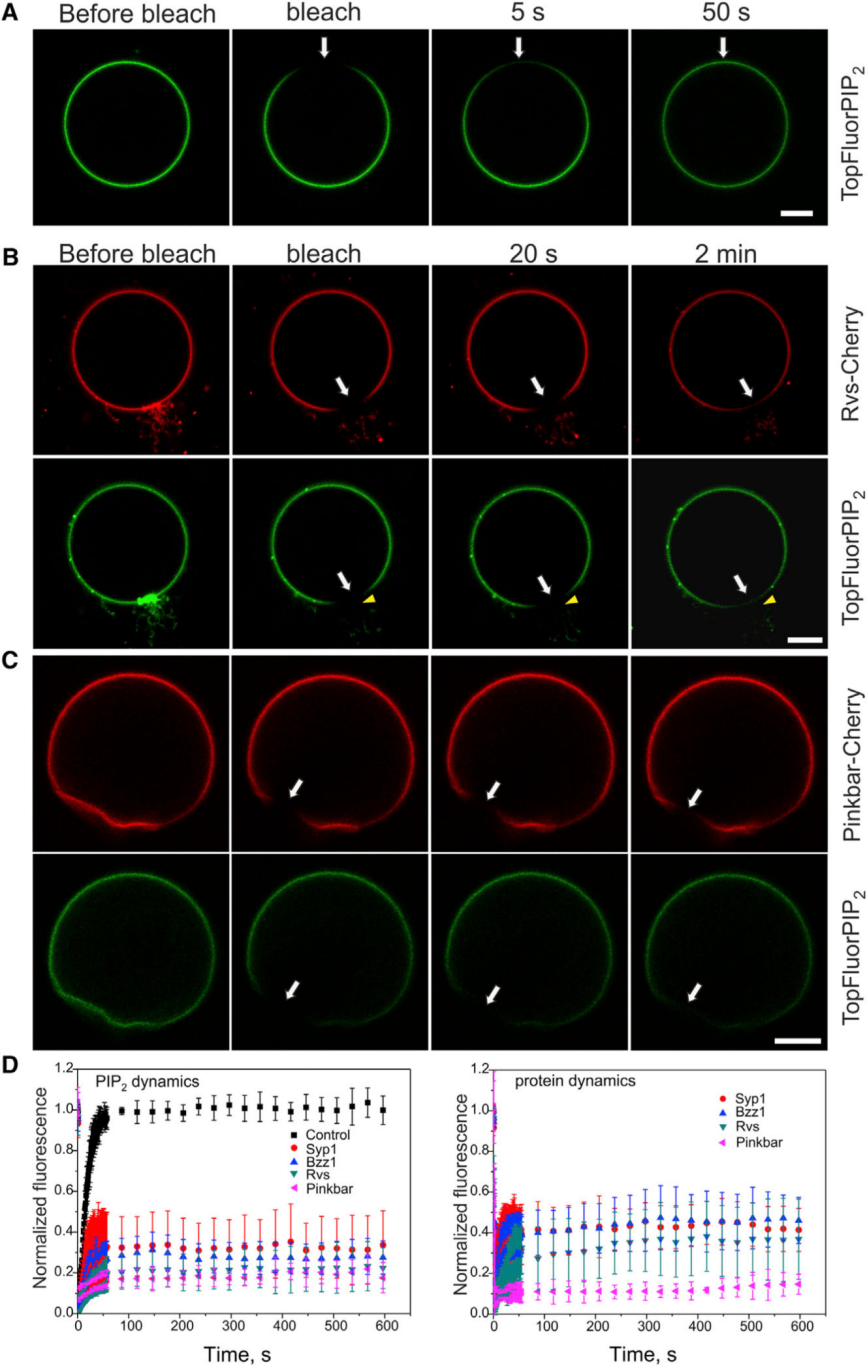
BAR Domains Induce the Formation of Stable Lipid Microdomains by Significantly Diminishing the Lateral Diffusion of PI(4,5)P_2_ (A) In a control vesicle, rapid recovery of TopFluor-PI(4,5)P_2_ was observed during the 50 s period following photobleaching. (B) Both PI(4,5)P_2_ and associated proteins displayed very slow recovery in membrane clusters and tubular regions induced by the Rvs161/167 BAR domain. Although partial recovery could be observed in the planar region of the photobleached area 2 min after the photobleaching, no recovery was detected at the membrane tubules. Please note that the unbleached tips of the tubules remain green during the recovery period, but the green TopFluor-PI(4,5)P_2_ does not detectably diffuse into the BAR domain-induced membrane tubules during the recovery period (arrowhead). Thus, the PI(4,5)P_2_ molecules do not display detectable diffusion within the BAR domain-induced membrane tubules. (C) The BAR domain of Pinkbar and PI(4,5)P_2_ displayed very slow recovery on planar membranes, suggesting that the BAR domain of Pinkbar forms stable scaffolds on membrane that efficiently decrease the diffusion of phosphoinositides. (D) Quantification of the recovery of TopFluor-PI(4,5)P_2_ and protein fluorescence in control vesicles in BAR/F-BAR domain cluster/tubule, as well as in planar membranes induced by the BAR domain of Pinkbar. In each case, the data are mean of at least five independent experiments and the error bars indicate ± SD. Further information on the analysis of FRAP data can be found in Figure S6. The lipid composition was POPC: POPE:POPS:PI(4,5)P_2_:TopFluor-PI(4,5)P_2_ = 50: 20:20:9:1. The protein concentrations were 1 µM. The scale bar represents 10 µM.

The plasma membrane of living cells is organized into specific heterogeneous domains with distinct protein and lipid compositions. However, the degree to which membrane-attached proteins can affect the lateral diffusion of lipids is poorly understood. For example, the possible effects of BAR domain proteins on lipid dynamics have not been reported. To examine whether BAR and F-BAR domains can significantly affect PI(4,5)P_2_ dynamics in the membrane, we carried out fluorescence recovery after photobleaching (FRAP) analysis of GUVs in the presence of fluorescently conjugated lipids and proteins. In the control vesicles, TopFluor-PI(4,5)P_2_ displayed fast recovery at the photobleached region, consistent with rapid lateral diffusion of lipid molecules (Figure 3A). Strikingly, both PI(4,5)P_2_ and the associated proteins displayed extremely slow recovery in the membrane clusters and tubular regions induced by the BAR/F-BAR domains of Syp1, Bzz1, and Rvs161/167 (Figures 3B and 3D). Furthermore, in the cases where only a segment of the membrane tubule was photobleached, the TopFuor-PI(4,5)P_2_ probe did not display redistribution within the tubule during the recovery period (arrowheads in Figure 3B). These results provide evidence for an almost complete lack of lateral diffusion of PI(4,5)P_2_ in the BAR domain-induced membrane tubules. To exclude the possibility that the diminished lateral diffusion of PI(4,5)P_2_ in membrane tubules is caused by membrane curvature generated by these proteins, we measured the protein dynamics and lateral diffusion of PI(4,5)P_2_ on planar membranes induced by the BAR domain of Pinkbar (Figures 3C and 3D). Similar to endocytic BAR and F-BAR domains, the BAR domain of Pinkbar and PI(4,5)P_2_ displayed very slow recovery on planar membranes, suggesting that severely reduced diffusion of phosphoinositides associated with the BAR domain scaffolds is not dependent on membrane curvature.

It is, however, important to note that efficient inhibition of lipid diffusion is dependent on protein density and oligomerization. This is because, at nontubular regions of the Rvs161/167-, Bzz1-, and Syp1-containing vesicles, where the protein was presumably not assembled into stable scaffolds, the lateral diffusion of lipids was only mildly affected (Figures S6A and S6B). Furthermore, a BAR domain mutant of Pinkbar (W141S) that is less efficient in forming oligomers (Pykäläinen et al., 2011) displayed less pronounced effects on phosphoinositide dynamics as compared to the wild-type BAR domain of Pinkbar (Figure S6D). It is also important to note that, in the protein/PI(4,5)P_2_ clusters, ~15%–35% of the lipid molecules displayed rapid diffusion (Figures S6F and S6G). This rapidly recovering PI(4,5)P_2_ population may correspond to those lipid molecules in the photobleached region that are not associated with BAR domain scaffolds. By excluding the rapidly recovering population, only extremely slow recovery of the remaining lipid molecules was detected at the photobleached region, suggesting that lateral diffusion of lipids in the BAR/F-BAR domain-induced membrane tubules is reduced by at least two orders of magnitude (Figures 3D, S6F, and S6G).

**Figure 4:**
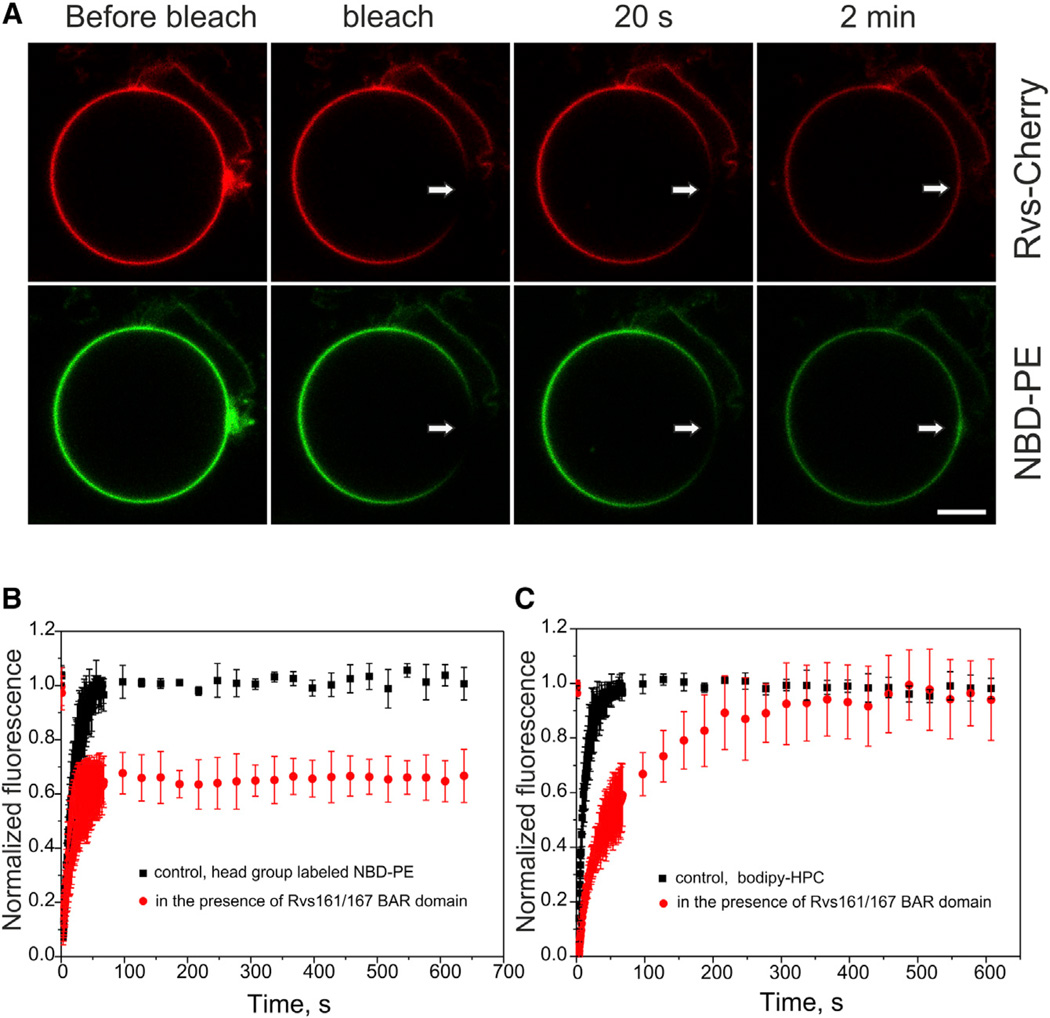
The F-BAR/BAR Domains of Syp1, Bzz1, and Rvs161/167 Inhibit the Lateral Diffusion of Zwitterionic Lipid PE in the Membrane Clusters and Tubules (A) A FRAP assay measuring the diffusion of NBD head group-labeled PE revealed that the lateral diffusion of this zwitterionic lipid was diminished in membrane clusters/tubules induced by the Rvs161/167 BAR domain. (B) Quantification of NBD-PE fluorescence recovery in control vesicles and in Rvs161/167 BAR domain-induced membrane tubules/clusters. It is important to note that a smaller fraction of NBD head group-labeled PE compared to PI(4,5)P_2_ (~40% versus 75%) displayed very slow lateral diffusion in BAR domain-induced structures. This is most likely due to less extensive enrichment of PE in BAR domain-induced membrane tubules compared to PI(4,5)P_2_ (see Figure 2D). (C) Dynamics of the acylchain labeled PC displayed only approximately 5-fold decrease in lateral diffusion in BAR domain clusters compared to control vesicles, and unlike head group labeled PE, it reached full fluorescence recovery during the 10 min monitoring period. In all cases, the data are mean of at least five independent experiments and the error bar indicates ± SD. The lipid composition was POPC:POPE:POPS:PI(4,5) P_2_:NBD-PE/BODIPY-HPC = 50:19:20:10:1. The concentration of the BAR domain of Rvs161/167 was 1 µM. The scale bar represents 10 µM.

We next examined the effects of the Rvs161/167 BAR domain on the dynamics of other lipid species, which do not concentrate to BAR domain scaffolds. FRAP assays using nitrobenzoxadiazole (NBD) head group-labeled PE revealed that the lateral diffusion of this zwitterionic lipid was also severely diminished in membrane clusters/tubules induced by the Rvs161/167 BAR domain, because ~40% of the fluorescence did not recover during the monitoring period (Figures 4A and 4B). In order to examine the possible effects of the relatively large artificial head group on the diffusion of NBD head group-labeled PE, we studied the dynamics of acyl chain-labeled PE and phosphatidylcholine (PC). Importantly, these acylchain-labeled lipids displayed only approximately 5-fold decreased dynamics in the BAR domain clusters as compared to control vesicles, and unlike head group-labeled PE, they reached full fluorescence recovery during the 10 min monitoring period (Figures 4C and S6E). Thus, although BAR domain scaffolds can efficiently inhibit the dynamics of PI(4,5)P_2_, they display significantly smaller effects on the lateral diffusion of nonbound lipids.

Collectively, these data demonstrate that the budding yeast endocytic BAR and F-BAR domains and the BAR domain of mouse Pinkbar can assemble into very stable scaffolds on membranes. By efficiently inhibiting the lateral diffusion of PI(4,5)P_2_, the BAR domain scaffolds can generate extremely stable lipid microdomains.

### Eisosomal BAR Domain Protein Lsp1 Forms Stable Scaffolds In Vitro and in Cells and Efficiently Inhibits the Lateral Diffusion of Lipids

**Figure 5:**
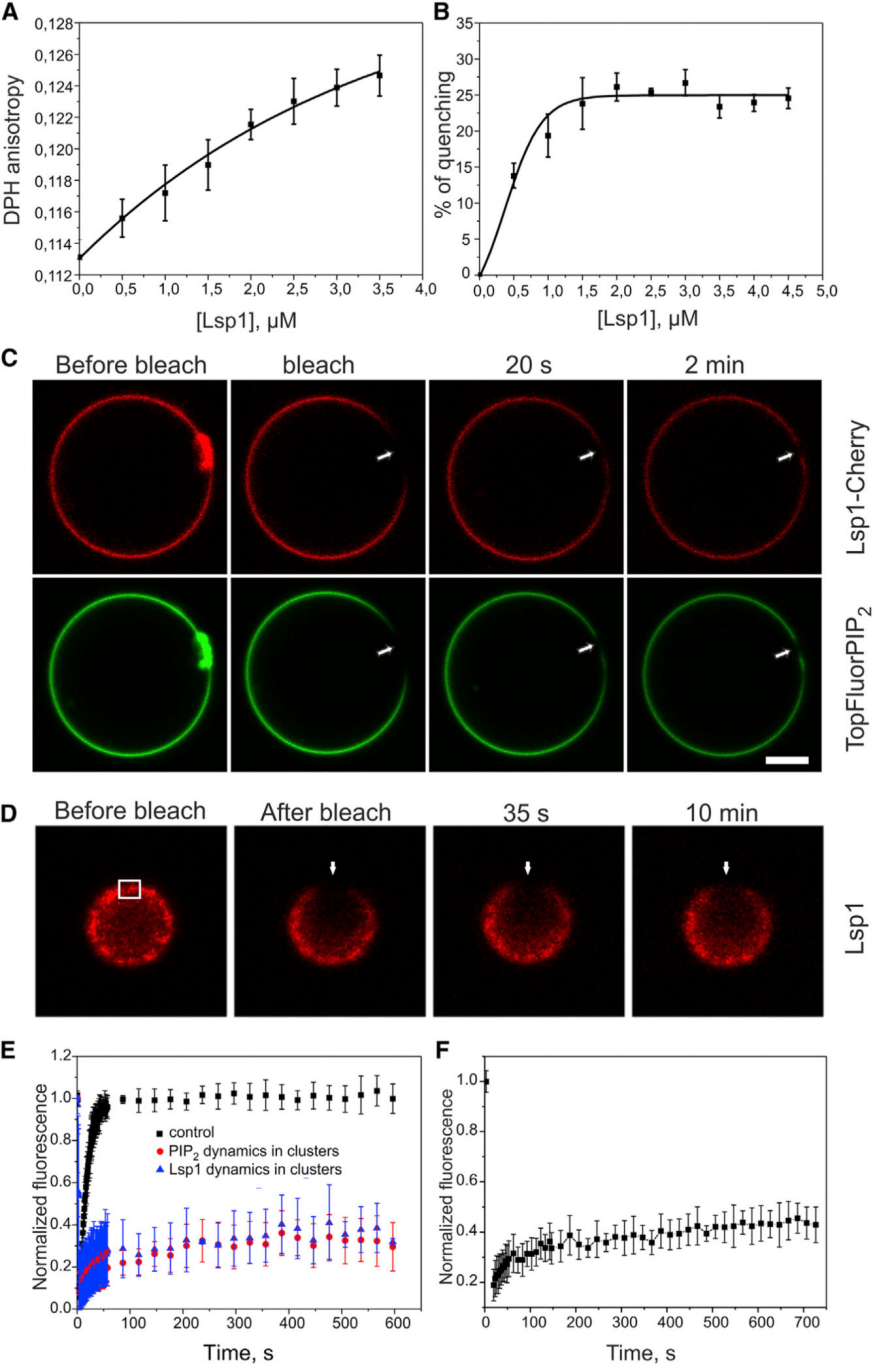
The BAR Domain of Eisosomal Protein Lsp1 Forms Stable Membrane Microdomains, and the Protein Displays Slow Dynamics at the Plasma Membrane of Yeast Cells (A) The BAR domain of Lsp1 decreased membrane fluidity in a concentration-dependent manner, as indicated by the increase in steady-state DPH anisotropy. This suggests that the domain not only interacts with the lipid head groups but also penetrates into the acyl-chain region of the bilayer. The lipid composition was POPC:POPE:POPS: PI(4,5)P_2_ = 50:20:20:10. DPH was incorporated at 1/500 ratio, and the lipid concentration was 40 µM. (B) The Lsp1 BAR domain efficiently induced the clustering of PI(4,5)P_2_, as measured by the self-quenching of BODIPY-TMR-PI(4,5)P_2_. The lipid composition was as described in Figure 2A. (C) FRAP analysis on GUVs revealed that the BAR domain of Lsp1 forms stable protein scaffolds and efficiently inhibits the lateral diffusion of PI(4,5)P_2_. The scale bar represents 10 µM. The lipid composition and protein concentration were as described in Figure 3. (D) In agreement with the biochemical data, full-length Lsp1 displayed similar slow dynamics at the plasma membrane of yeast cells. (E) Quantification of the fluorescence recovery of TopFluor-PI(4,5)P_2_ and the BAR domain of Lsp1 in membrane clusters/tubules. Furthermore, quantification of the fluorescence recovery of TopFluor-PI(4,5)P_2_ in control vesicles in the absence of the BAR domain is shown. The values in the graph are mean of at least five independent experiments, and the error bar represents ± SD. (F) Quantification of the fluorescence recovery of full-length Lsp1 at the plasma membrane of yeast cells. The values in the graph are mean of ten independent FRAP experiments, and the error bars represent ± SD.

Endocytosis is a dynamic process, which requires a large number of proteins. The membrane-bound scaffolds formed by the endocytic BAR/F-BAR domain proteins are transient, and their assembly and disassembly during this process are precisely regulated (Henne et al., 2010; Kaksonen et al., 2005; Taylor et al., 2011). However, budding yeast also harbors another structurally similar plasma membrane invagination structure, which is very stable. These structures, called eisosomes, may function in lipid storage and homeostasis (Walther et al., 2006; Ziółkowska et al., 2012). The membrane invaginations in eisosomes are primarily generated by two BAR domain proteins, Pil1 and Lsp1, which can assemble into similar oligomeric helical scaffolds to the endocytic BAR and F-BAR domains (Frost et al., 2008; Kabeche et al., 2011; Karotki et al., 2011; Mim et al., 2012; Olivera-Couto et al., 2011; Ziółkowska et al., 2011). Thus, we examined the effects of the Lsp1 BAR domain on the organization and dynamics of phosphoinositides in vitro. Similarly to the BAR domain of Rvs161/167, the BAR domain of Lsp1 decreased membrane fluidity, indicating that, in addition to interaction with the lipid head groups, it penetrates into the acylchain region of the bilayer (Figure 5A). Lsp1 BAR domain also promoted the quenching of BODIPY-conjugated PI(4,5)P_2_ in a concentration-dependent manner, indicating that it induces the clustering of PI(4,5)P_2_ (Figure 5B). Importantly, FRAP analysis on GUVs revealed that the BAR domain of Lsp1 forms stable scaffolds, which efficiently inhibit the lateral diffusion of PI(4,5)P_2_ (Figures 5C and 5E). FRAP analysis on budding yeast cells expressing an RFP fusion of the full-length Lsp1 protein revealed that this protein also assembles into similar stable clusters at the plasma membrane of cells as on the GUVs in vitro (Figures 5D and 5F). Taken together, these data show that the eisosomal BAR domain scaffolds also generate extremely stable lipid microdomains in vitro. Importantly, full-length Lsp1 displays similar dynamics in vivo compared to its isolated BAR domain on GUVs, suggesting that this protein efficiently also diminishes the lateral diffusion of lipids at the plasma membrane of living cells.

## DISCUSSION

BAR superfamily proteins are key regulators of plasma membrane morphology and contribute to a wide range of cellular processes, ranging from endocytosis to cell migration and adhesion (Qualmann et al., 2011; Zhao et al., 2011). BAR domains sculpt membranes to generate plasma membrane protrusions and invaginations, but whether these proteins can also affect other physicochemical properties of membranes has not been reported. Strikingly, our data revealed that the protein scaffolds formed by BAR superfamily domains “freeze” lipid dynamics by nearly completely inhibiting the lateral diffusion of phosphoinositides and can thus generate extremely stable protein-lipid microdomains.

Generation of stable lipid microdomains appears to be a general and specific feature of all BAR domain proteins. This is because all BAR domains tested here generated extremely stable phosphoinositide microdomains, whereas a PI(4,5)P_2_-binding pleckstrin homology (PH) domain did not display detectable effects on the lateral diffusion of phosphoinositides (Figure S6C). Importantly, membrane curvature alone is not responsible for diminished lipid diffusion in these structures, but BAR domain interactions with the lipid head groups play an important role. This is because, although lateral diffusion of lipid molecules in tubular (diameter 10 nm) membranes is ~2- to 3-fold slower compared to planar membranes (Domanov et al., 2011), the diffusion of phosphoinositides in the BAR/F-BAR domain -induced membrane tubules (with a diameter of ~20 nm) was at least two orders of magnitude slower compared to control membranes. Furthermore, the lateral diffusion of PI(4,5)P_2_ is dramatically diminished on planar membranes induced by the BAR domain of Pinkbar (Figures 3C and 3D). Thus, assembly of elongated BAR and F-BAR domains, which interact with phosphoinositides through a multivalent manner (Figures 2 and 5B), into helical scaffolds around membrane tubules (Frost et al., 2008; Mim et al., 2012) or as a sheet on a flat membrane (Pykäläinen et al., 2011) will severely limit the diffusion of individual phosphoinositide molecules. The effects of BAR domains on the lateral diffusion of phosphoinositides are strongly dependent on protein density and seem to require the assembly of oligomeric BAR domain scaffolds. In agreement with this conclusion, a recent study proposed that the amphiphysin-1 BAR domain functions as a membrane curvature sensor at low protein density, whereas a high local density of the domain is required for its ability to oligomerize and induce membrane tubules (Sorre et al., 2012).

BAR domains may also have profound effects on membrane dynamics in cells. The eisosomal BAR domains Pil1 (Brach et al., 2011; Kabeche et al., 2011) and Lsp1 (Figure 5) form very stable plasma membrane-associated scaffolds in yeast cells, which are expected to display similar effects on lipid-dynamics, as we observed for Lsp1 scaffolds in vitro. Although the BAR domain scaffolds in endocytosis are precisely controlled and transient, these proteins are capable in forming very stable structures in cells if their regulation is disturbed. For example, a recent study revealed that a small molecule inhibition of the clathrin function resulted in the formation of very stable FCHo2 F-BAR domain scaffolds in mammalian cells (von Kleist et al., 2011) and that F-BAR domain protein FBP17 displays slow turnover in membrane tubules in a cell-free system (Wu et al., 2010). Furthermore, previous studies suggested that the diffusion of lipids at the plasma membrane varies significantly between different surface domains (Wolfe et al., 1998) and that distinct PI(4,5)P_2_ pools are present at the inner leaflet of the plasma membrane (Golebiewska et al., 2008). Finally, the lateral diffusion of PI(4,5)P_2_ in cardiomyocyte membranes and at the plasma membrane of pathogenic fungus *Candida albicans* were reported to be surprisingly slow (Cho et al., 2005; Vernay et al., 2012), suggesting that, in living cells, the dynamics of lipids at the plasma membrane are greatly affected by interactions with various membrane-binding proteins, including the BAR domain scaffolds.

**Figure 6:**
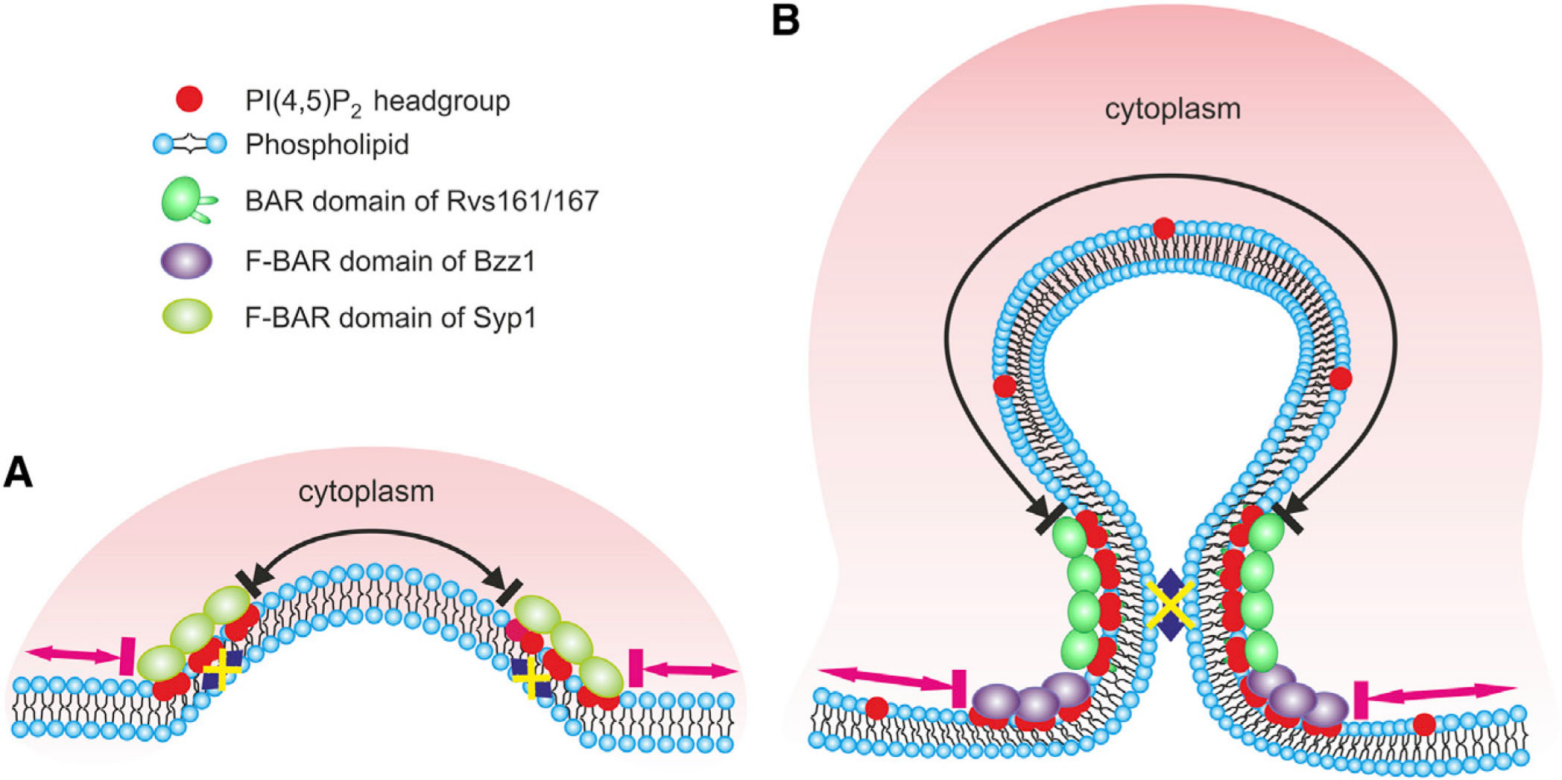
A Schematic Model of the Effects of F-BAR/BAR Domains on the Distribution and Dynamics of PI(4,5)P_2_ in Endocytic Invaginations (A) Syp1 is the first BAR superfamily protein to arrive at the sites of endocytosis in budding yeast. Although the F-BAR domain of Syp1 does not display specificity for PI(4,5)P_2_, it efficiently inhibits the lateral diffusion of phosphoinositides in the clusters (blue arrow). Thus, the Syp1 oligomer may form a lipid diffusion barrier between the tip of the invagination (black arrow) and the surrounding regions of the plasma membrane (pink arrows). (B) During the subsequent phase of endocytosis, Bzz1 binds at the invagination base through its F-BAR domain to stabilize the endocytic site with Rvs161/167, which localizes along the membrane tubule through its BAR domain. The Bzz1-Rvs161/167 scaffold efficiently inhibits the lateral diffusion of PI(4,5)P_2_ in this region (blue arrow) and may thus be involved in vesicle scission. The phosphoinositides and membrane proteins outside this region can diffuse freely (pink arrows) but cannot enter to the neck region, due to a lipid diffusion barrier formed by Bzz1 and Rvs161/167. Similarly, phosphoinositides and membrane proteins can diffuse within the tip of the endocytic bud (black arrow) but cannot exit the site, due to the lipid diffusion barrier.

Generation of stable membrane microdomains by BAR superfamily proteins may have an important biological role in various processes. Inhibition of lateral diffusion of phosphoinositides is expected to generate lipid-phase boundaries at both ends of the BAR domain scaffold. These sites may act as hot spots for vesicle scission in endocytosis, as proposed by Liu et al. (2009), because of high line tension at domain boundaries. However, a recent study provided evidence that insertion of amphipathic motifs to the bilayer would drive vesicle scission during endocytosis (Boucrot et al., 2012). These two possible vesicle scission mechanisms are not necessarily mutually exclusive. Thus, future studies are needed to elucidate their relative contributions, combined with the force provided by actin polymerization, to vesicle scission during endocytosis. In addition to the role in vesicle scission, the stable membrane microdomains induced by BAR domains are expected to function as lipid diffusion barriers. Our data show that BAR domain scaffolds efficiently inhibit the diffusion of PI(4,5)P_2_ without drastically affecting the dynamics of those lipid species (e.g., PE and PC) that do not display specific interactions with the BAR domain scaffold. Furthermore, we propose that the stable BAR/F-BAR domain scaffolds and the underlying PI(4,5)P_2_ microdomains may limit the diffusion of transmembrane proteins and cytoplasmic membrane-anchored proteins. This could be important for trapping certain membrane proteins at the tip of the endocytic bud, as well as for preventing the entry of other membrane anchored molecules into this region (see Figure 6), although additional in vitro and in vivo work is required to reveal the exact organization and function of BAR domain proteins at the endocytic sites. Thus, BAR superfamily proteins may form diffusion barriers in smaller scale structures compared to septins, which are membrane-associated proteins that form diffusion barriers, for example, at the primary cilia and at the neck of budding yeast cells (Saarikangas and Barral, 2011). In the case of eisosomes, the stable BAR domain-induced tubular lipid microdomains may be important in storage of lipids and membrane-anchored proteins.

Our data also demonstrate that the BAR/F-BAR domains of Syp1, Bzz1, and Rvs161/167 display significant differences in their lipid specificities, as well as in interactions with the acylchain region of the lipid bilayer. These differences may at least partially account for the specific roles of the three proteins during distinct steps of the endocytic internalization process. For example, clear PI(4,5)P_2_ specificity of the Rvs161/167 BAR domain heterodimer compared to Syp1 F-BAR domain is in line with the increase in PI(4,5)P_2_ levels in conjunction with coat and actin assembly during endocytosis in budding yeast (Sun et al., 2007; Sun and Drubin, 2012). It is, however, important to note that Syp1, Bzz1, and Rvs161/167 are relatively large multidomain proteins that also harbor other interaction motifs. Syp1, for example, contains another short membrane-binding motif following the F-BAR domain (Reider et al., 2009). Thus, these additional motifs are likely to affect the membrane-binding properties of the full-length proteins and to thus introduce further complexity to the membrane interaction mechanisms of these three endocytic BAR/F-BAR domain proteins. Interestingly, although only the Rvs161/167 BAR domain displayed clear preference toward vesicles with high PI(4,5)P_2_ density, the F-BAR domains of Syp1 and Bzz1 can also induce PI(4,5)P_2_ clustering in vitro (Figures 1A and 2A). Structurally, this may be explained by much smaller positively charged phosphoinositide-binding interface on the F-BAR domain of Syp1 compared to the BAR domain of Rvs161/167 (Reider et al., 2009). This site is expected to induce phosphoinositide clustering at low PI(4,5)P_2_ densities, whereas higher PI(4,5)P_2_ density on the membrane could induce repulsion forces between negatively charged phosphate groups of PI(4,5)P_2_ and the neutral/negatively charged amino acids outside the phosphoinositide-binding pocket of Syp1. It is, however, also important to note that the self-assembly mechanisms of N-BAR and F-BAR domains are different. Protein scaffolds formed by endophilin N-BAR domain are held together through interactions between endophilin’s amphipathic N-terminal helices, whereas the F-BAR domain scaffolds are stabilized through lateral contacts between the coiled-coil regions of the domains (Frost et al., 2008; Mim et al., 2012). Importantly, membrane insertion of the N-terminal a helix of Rvs161/167, endophilin, and amphiphysin BAR domains is enhanced by PI(4,5)P_2_ (Figures S3E and S3F; Yoon et al., 2012), demonstrating that, in addition to the coiled-coil region, also the N-terminal helix contributes to phosphoinositide specificity of N-BAR domains.

In conclusion, this work provides evidence that, in addition to their membrane curvature sensing/generating activity, BAR domains also induce the formation of extremely stable membrane microdomains. The phosphoinositide microdomains induced by the membrane-associated BAR domain scaffolds are distinct from classical lipid rafts, which arise through preferential association of sterols, sphingolipids, and specific integral membrane proteins (Lingwood and Simons, 2010). Although our experiments with the eisosomal BAR domain protein Lsp1 suggested that BAR domains can also form stable membrane scaffolds in vivo to limit lipid diffusion, future studies will be needed to elucidate the extent to which BAR domains affect lateral diffusion of lipids in cells and the biological roles of this activity. These studies will require development of new methods that would allow the dynamics of specific lipid species in living cells to be studied. Moreover, future studies are needed to reveal the exact nature of the BAR/F-BAR domain scaffolds at the neck of endocytic invaginations, and to uncover how these scaffolds affect the diffusion of membrane proteins during endocytosis.

## EXPERIMENTAL PROCEDURES

### Lipid-Binding Assays

The lipid-binding assays were performed as described in Saarikangas et al. (2009). The tryptophan fluorescence assay for examining the interaction of Bzz1 F-BAR domain with vesicles was carried out as described in Zhao et al., (2010). In the replacement cosedimentation assay, 1 µM Syp1 F-BAR domain was incubated with 250 µM liposomes for 10 min followed by the addition of the BAR domain of Rvs161/167. The sample was incubated for 10 min at room temperature, and the vesicles and bound proteins were sedimented by centrifugation.

### Homo-FRET anisotropy of Rvs-Cherry

The steady state homo-FRET anisotropy of Rvs-Cherry was measured by Perkin-Elmer LS 55 spectrometer. Rvs161 was tagged with Cherry at its C terminus. The heterodimeric Rvs161/167 BAR domain was excited at 585 nm and emission was set at 604 nm, with both bandwidths of 5 nm. The concentration of Rvs-Cherry was 0.5 µM. Homo-FRET anisotropy was not applied for Syp1 and Bzz1 F-BAR domains, because they are homodimers and thus contain two closely connected cherry molecules already in solution.

### FRAP and Live-Imaging of GUVs

Imaging of GUVs was performed on a confocal microscope (Leica TCS SP5) equipped with Leica Confocal Software. A HCX PL APO 63X/1,2 W Corr/0,17 CS (water) Lbd. bl. objective was used for all experiments. In the GUV binding assay, the protein concentration was 1 µM. In the Syp1-GFP and Rvs161/ 167-Cherry competition assay, both proteins were premixed at equal concentration and added to the GUV at a final concentration of 1 µM. GFP, TopfluorPIP_2_, and NBD-PE were imaged using a 488 nm laser line, and Cherry-fusion proteins were imaged using a 561 nm laser line. Three pre-bleach images were taken, and after which, five bleaching scans (3.9 s each) with 100% intensity of 488 nm laser lines over the region of interest were performed. Recovery of fluorescence was monitored ten times every 3.9 s, 60 times every 1 s, and 20 times every 30 s. The intensity of the bleached area was normalized to the nonbleached GUV fluorescence intensity to diminish error caused by normal photobleaching during the monitoring period. The value before bleach was normalized to 1.0, and mean plots were calculated from five to nine different FRAP experiments. All the error bars indicate ± SD. The data were fitted with SigmaPlot 11.0 to a single f = a * (1 – exp(−b * x) exponential equation. Mobile fractions and recovery half-times (t_1/2_) were obtained for each recovery curve, and the means and standard deviations were calculated.

## Supplementary Material

01
