# Supplementary material for: Membrane-Sculpting BAR Domains Generate Stable Lipid Microdomains
Source: Cell Rep. Author manuscript; Available in PMC 2014 Sep 26. (PMC4105227; doi:10.1016/j.celrep.2013.08.024)
Supplement: 01 [file NIHMS605461-supplement-01.pdf]

Supplemental Information

Cell PRESS Open ACCESS

EXTENDED EXPERIMENTAL PROCEDURES

Materials

DiO-C18, Neutravidin, 1,6-diphenyl-1,3,5-hexatriene (DPH), β-bodipy®500/510 C12-HPC and *N*-NBD-PE (*N*-(7-Nitrobenz-2-Oxa-1,3-Diazol-4-yl)-1,2-Dihexadecanoyl-*sn*-Glycero-3-Phosphoethanolamine) were obtained from Invitrogen. POPC, POPE, POPS, and PI(4,5)P_2, DOPE-biotin, TopFluor PI(4,5)P_2, and acyl chain labeled NBD-PE (1-oleoyl-2-{12-[(7-nitro-2-1,3-benzoxadiazol-4-yl) amino] dodecanoyl}-***sn***-glycero-3-phosphoethanolamine) were purchased from Avanti Polar Lipids (Alabaster, AL). Bodipy-TMR-PI(4,5)P_2 was purchased from Echelon Biosciences (Salt Lake City, Utah). PLL-g-PEG2000 and PLL-g-PEG3400-biotin were purchased from Surface Solutions (Zurich, Schwitzerland). D-sorbitol was from Sigma-Aldrich (Brøndby, Denmark).

Media and Strains

The yeast strain used was H3463, LSP1::LSP1-Rfp-KanMX leu2-3, 112 ura3-52 (kindly provided by Dr. Jussi Jäntti's lab, Institute of Biotechnology, University of Helsinki, Finland). For fluorescence microscopy, yeast cells were grown to mid-log phase in SC-Trp medium at 30°C. Cells were mounted in the same media on glass-bottom microplates previously coated with polylysine and directly imaged with a Leica TCS SP5 confocal microscope.

Plasmid Construction

DNA fragments corresponding to gene-coding sequences were obtained by PCR amplification (Phusion High-Fidelity DNA Polymerase, Finnzymes) of yeast genomic DNA, pFA6a-mCherry-kanMX6 and pET-28(+)-6xHis-sfGFP plasmids. To express Bzz1-, Syp1- and Lsp1-derived proteins, DNA fragments were cloned into pGEX2T plasmid between the BamHI and XmaI cloning sites (for untagged BAR/F-BAR expression), or between BamHI and SacI (for fluorescent protein tagged BAR/F-BAR expression) and SacI and EcoRI (for Cherry and GFP expression). For Rvs-derived proteins, DNA fragments were cloned into pRSFDuet-1 plasmid between BamHI and XmaI (for untagged Rvs161 expression) or between BamHI and SacI (for fluorescent protein tagged Rvs161 expression) and SacI and HindIII (for Cherry and GFP expression), and between BglII and EcoRV (for Rvs167-BAR expression). Inserts were sequenced to confirm the absence of mutations. A DNA fragment for expressing mCherry-fusion of PLCδ PH domain (residues of PH domain with a C-terminally fused mCherry) was cloned into pHAT vector between SpeI and HindIII.

Protein Production

Bzz1, Syp1 and Lsp1-derived proteins were expressed using BL21(DE3)pLysS cells (EMD Chemicals) at 20°C for 18h. For Rvs-derived proteins, protein expression was induced at 37°C for 3h30. For GST-tagged protein affinity purification (Bzz1, Syp1 and Lsp1-derived proteins), pellets were resuspended in HK buffer (50 mM HEPES pH 7.5; 150 mM KCl) supplemented with protease inhibitors (Complete, Roche). Cells were lysed by sonication and the extract was centrifuged for 15 min at 90,000 g. Supernatants were incubated for 30 min at room temparature with Gluthatione Sepharose 4B (GE Healthcare Life Sciences). Bound proteins were batch bound to the resin in HK buffer, eluted with 10 units of thrombin (Sigma), concentrated (Amicon Ultra, 10k, Millipore) and dialyzed overnight against HKG buffer (50 mM HEPES, pH 7.5, 150 mM KCl, 5% glycerol). For His-tagged protein affinity purification (Rvs-derived proteins), pellets were resuspended in HKI1 buffer (50 mM HEPES, pH 7.5, 500 mM KCl, 5 mM Imidazole pH 7.5) supplemented with protease inhibitors (Complete, Roche). Cells were lysed by sonication and centrifuged for 15 min at 90,000 g. Supernatants were processed 10 times through a 22-gauge needle to sever bacterial DNA, and passed through a HisTrap HP column (GE Healthcare Life Sciences). Bound proteins were purified with HKI1 buffer, eluted with a gradient of HKI2 buffer (50 mM HEPES, pH 7.5, 500 mM KCl, 500 mM Imidazole pH 7.5), concentrated (Amicon Ultra, 10k, Millipore) and dyalized overnight against HKG buffer (50 mM HEPES, pH 7.5, 150 mM KCl, 5% glycerol). mCherry-fusion of PLCδ PH domain was expressed at 16°C for 16 hr. The protein was enriched with Ni-NTA Superflow beads (Sigma-Aldrich), and further purified by gel filtration using Superdex 75 column (GE Healthcare). Wild-type and W141S mutant BAR domain of Pinkbar were purified as described (Pykäläinen et al., 2011).

Preparation of Vesicles

Large unilamellar vesicles (LUVs) and giant unilamellar vesicles were prepared as described (Saarikangas et al., 2009). LUVs with a diameter of ~100 nm were used for all the fluorescence measurements, 1000 nm for curvature sensing experiment, and ~400 nm for the negative staining electron microscopy experiments.

Negative-Stain Electron Microscopy

Samples for transmission electron microscopy were prepared by mixing 10 μM proteins with 200 μM unilamellar vesicles (with a diameter of ~400 nm) in 20 mM HEPES, pH 7.5, 100 mM NaCl at room temperature for 15 min. The mixture was applied to the glow-discharged collodion- and carbon-coated copper grids and stained with 3% uranyl acetate. At each step, excess solution was removed by filter paper. The membrane morphologies were examined with an electron microscope (Tecnai 12 FEG; FEI Corp.) operating at 80 kV. Images were recorded with a 4k × 4k Ultrascan 4000 CCD camera (Gatan Corp.) with a magnification of 11000- 18500.

The Single Liposome Curvature Assay

The assay was carried out as previously described (Bhatia et al., 2009). In brief, liposomes (POPC:POPE:POPS:PIP_2:DOPE-biotin:DiO-C18 = 55:19:20:5: 0.5:0.5.) were given 30 min to immobilize to the functionalized surface, after which the remaining unbound liposomes were removed by washing. The protein solutions were carefully added to the chamber. The sample was left to incubate for 15 min before imaging. All experiments were performed using a Leica TCS SP5 inverted confocal microscope using an oil immersion objective HCX PLAPO CS x 100 (NA 1.4). Laser light wavelengths of 488 nm and 594 nm were used to excite DiO-C18 and mCherry labels, respectively. Images were taken sequentially to avoid crosstalk, at 200 Hz and a resolution of 2048 × 2048 pixels, with an pixel size of 25 nm x 25 nm and a bit depth of 16 bit. Liposomes were imaged before protein addition to ensure that the proteins did not significantly affect liposomes morphology or fluorescence. Similarly, control experiments ensured that appropriate settings were used where cross talk and emission bleed through do not affect our measurements.

Bodipy and Brominated-Phospholipid Quenching Experiment

The fluorescence measurements were performed as described in (Saarikangas et al., 2009). All fluorescence measurements were performed in quartz cuvettes with 3 mm path length. The fluorescence spectra were measured with a Perkin-Elmer LS 55 spectrometer. Because divalent ions for example Ca^{2+} can induce clustering of phosphoinositides (Wang et al., 2012), these experiments were carried out in the absence of divalent cations in 20 mM HEPES, 100 mM NaCl, 0.1mM EDTA, pH 7.5 at room temperature.

Fluorescence Anisotropy of DPH

DPH is a fluorescence probe located in the hydrophobic core of a lipid bilayer and is applied to monitor changes in the rotational diffusion of acyl chains in the membrane interior (Zaritsky et al., 1985). The DPH anisotropy was carried out as described in (Saarikangas et al., 2009).

Laurdan Fluorescence Measurements

Laurdan is a fluorescent molecule that detects changes in membrane phase properties through its sensitivity to the polarity of the environment in the bilayer. Polarity changes are shown by shifts in the Laurdan emission spectrum, which are quantified by calculating the generalized polarization (GP) (Parasassi et al., 1990). The excitation wavelength was 350 nm and emission was monitored at 440 and 490 nm. The bandwidth was 5 nm for both excitation and emission. The laurdan emission generalized polarization (GP) was calculated using the equation $GP = (I_{440}-I_{490})/(I_{440}+I_{490})$, where I_{440} and I_{490} are the emission intensities measured at 440 and 490 nm. The experiments were carried out in 20 mM HEPES, 100 mM NaCl, pH 7.5 at room temperature. The laurdan/lipid ratio was 1:1000.

SUPPLEMENTAL REFERENCES

Hatzakis, N.S., Bhatia, V.K., Larsen, J., Madsen, K.L., Bolinger, P.Y., Kunding, A.H., Castillo, J., Gether, U., Hedegård, P., and Stamou, D. (2009). How curved membranes recruit amphipathic helices and protein anchoring motifs. Nat. Chem. Biol. *5*, 835–841.

Wang, Y.H., Collins, A., Guo, L., Smith-Dupont, K.B., Gai, F., Svitkina, T., and Janmey, P.A. (2012). Divalent cation-induced cluster formation by polyphosphoinositides in model membranes. J. Am. Chem. Soc. *134*, 3387–3395.

Zaritsky, A., Parola, A.H., Abdah, M., and Masalha, H. (1985). Homeoviscous adaptation, growth rate, and morphogenesis in bacteria. Biophys. J. *48*, 337–339.

Cell PRESS Open ACCESS

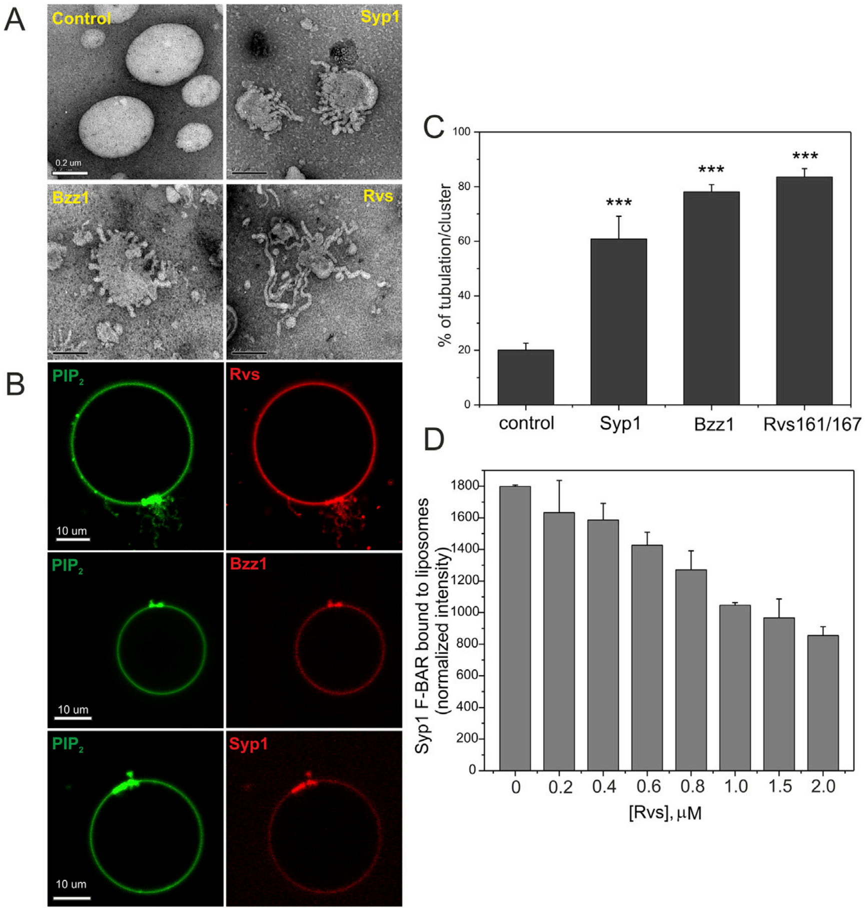

Figure S1. The F-BAR/BAR Domains of Syp1, Bzz1, and Rvs161/167 Induce Membrane Tubulation, Related to Results

(A) Electron microscopy analysis of membrane tubules induced by the F-BAR/BAR domains of Syp1, Bzz1 and Rvs161/167. These F-BAR/BAR domains deformed the PI(4,5)P_2 membrane into tubules with a diameter of 20 ± 2.5 nm. The vesicle diameter used in the Negative-Stain Electron Microscopy was 400 nm. The concentrations of proteins and lipids were 10 μM and 200 μM, respectively. The lipid composition was POPC:POPE:POPS:PI(4,5)P_2 = 50:20:20:10. Scale bar: 0.2 μm.

(B) Addition of Cherry-fusion F-BAR/BAR domains (Cherry was fused at the C terminus of the proteins) to vesicles induced efficient membrane tubulation as detected by fluorescence microscopy of GUVs (images shown were acquired 10 min after the addition of proteins). The composition of the GUVs was POPC:POPE:POPS:PI(4,5)P_2:TopFluorPI(4,5)P_2 = 50:20:20:9:1, and the concentration of the BAR domain was 1 μM. Scale bar: 10 μm.

(C) Quantification of the proportion of GUVs displaying membrane tubulation or PI(4,5)P_2-clustering in the absence (control) and presence of the F-BAR/BAR domains of Syp1, Bzz1, and Rvs161/167. In each case, 100-120 vesicles were examined. The error bars represent ± SD from three independent experiments. The difference between the control vesicles and protein-bound vesicles are statistically significant (***$p < 0.001$, student t test). The composition of the GUVs was POPC:POPE:POPS:PI(4,5)P_2:TopFluorPI(4,5)P_2 = 50:20:20:9:1, and the concentrations of the proteins were 1 μM.

(D) The BAR domain of Rvs161/167 replaces Syp1 F-BAR domain from PI(4,5)P_2-containing membranes as measured by a vesicle co-sedimentation assay. In this assay, 1 μM Syp1 F-BAR domain was incubated first with 250 μM liposomes for 10 min and then the BAR domain of Rvs161/167 was added. After incubation of 10 min the sample was centrifuged. The lipid composition was POPC:POPE:POPS:PI(4,5)P_2 = 50:20:20:10.

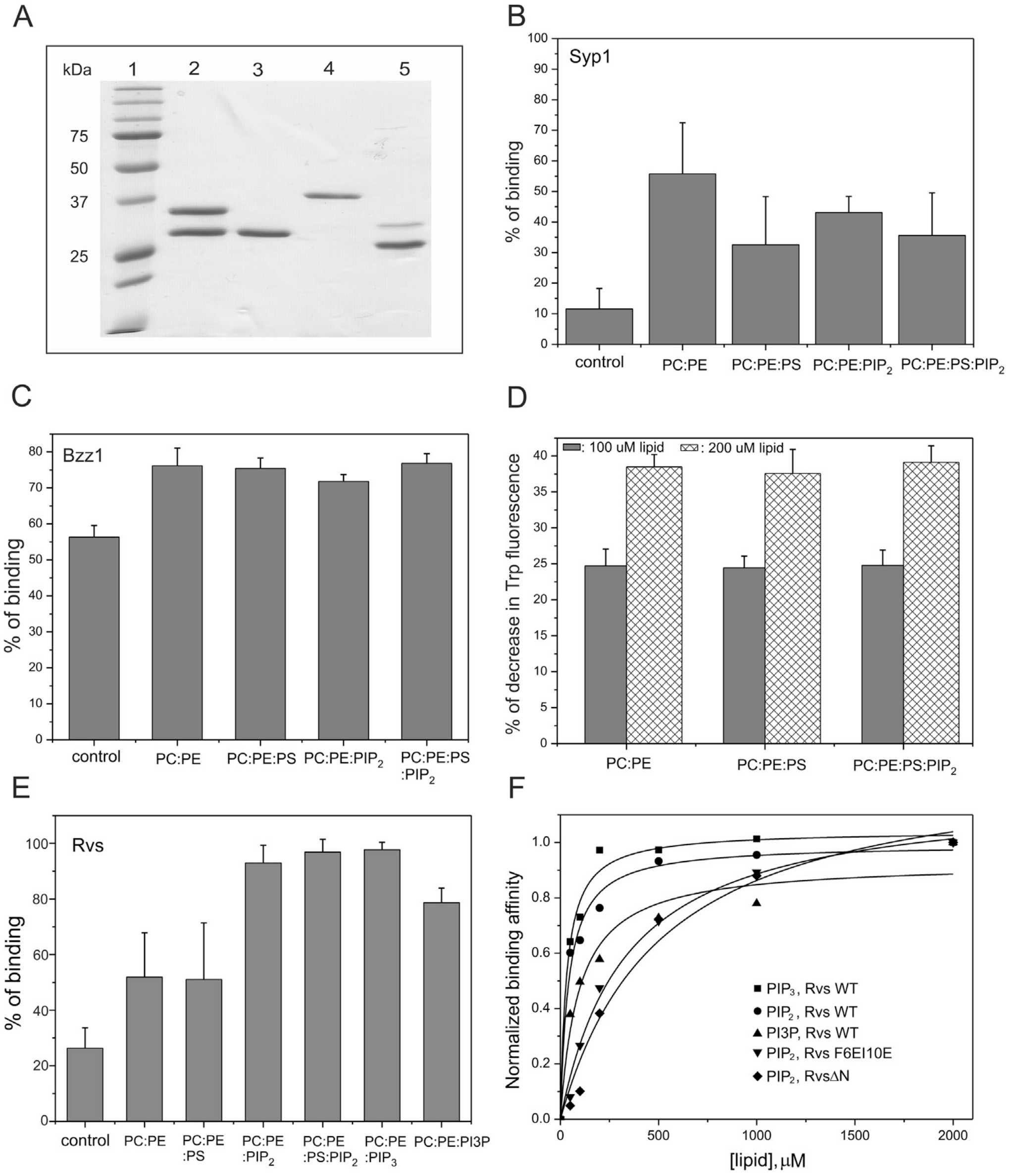

Figure S2. Lipid Specificities of the F-BAR/BAR Domains of Syp1, Bzz1, and Rvs161/167, Related to Results

(A) The monodispersity of the proteins used in the study. The lanes 1-5 represent the protein standard, heterodimeric Rvs161/167 BAR domain, Syp1 F-BAR domain, Bzz1 F-BAR domain, and Lsp1 BAR domain, respectively.

(B) Based on vesicle co-sedimentation assays, the membrane-binding of Syp1 F-BAR domain displayed no preference for PI(4,5)P_2. The protein and lipid concentrations in the co-sedimentation assay were 1 μM and l mM, respectively.

(C) Binding of the F-BAR domain of Bzz1 was not dependent on the lipid composition of the membrane.

(D) Similarly to the vesicle co-sedimentation assay, a tryptophan fluorescence assay also indicated that the membrane binding of Bzz1 F-BAR domain does not display detectable lipid-specificity. Here, the Trp fluorescence of the Bzz1 F-BAR domain was similarly quenched by addition of vesicles containing (100 mM and 200 mM) PC:PE, PC:PE:PS and PC:PE:PS:PI(4,5)P_2. The bars indicate mean of three independent co-sedimentation or tryptophan fluorescence assays, and the error bars represent ± SD.

(E) In contrast to Syp1 and Bzz1 domains, the membrane-binding of the BAR domain of Rvs161/167 was enhanced by different phosphoinositides with an affinity of PI(3,4,5)P3 ≈ PI(4,5)P_2 > PI3P.

(F) Membrane interaction of the BAR domain of Rvs161/167 was enhanced by increasing the negative charge of phosphoinisitides with an affinity of PI(3,4,5)P_3 > PI(4,5)P_2 > PI3P. Mutation or deletion on the N-terminus of the BAR domain of Rvs161/167 reduced the binding of this domain to phosphoinositide-rich membranes. The lipid composition was POPC:POPE:POPS: phosphoinositide = 50:20:20:10, with only the outer leaflet of the bilayer are accessible to proteins. The protein concentration in the assay was 1 μM.

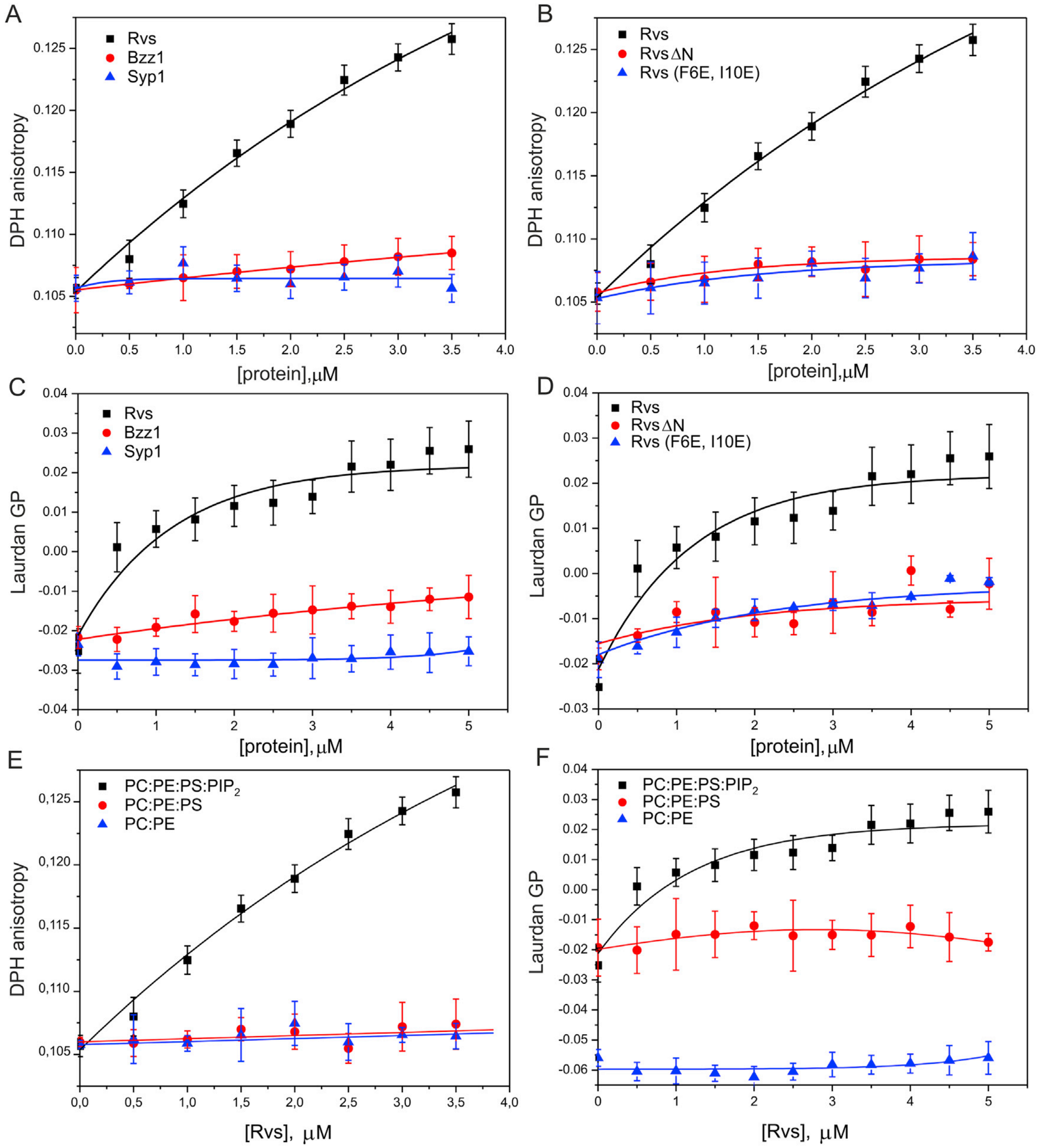

Figure S3. The BAR Domain of Rvs161/167 Decreases Membrane Fluidity and Increases Lipid Order, whereas the F-BAR Domains of Syp1 and Bzz1 Do Not Have Significant Effects on the Physiochemical Properties of the Membrane, Related to Results and Discussion

(A) The BAR domain of Rvs161/167 decreased the membrane fluidity as indicated by the increase in DPH anisotropy, suggesting that its inserts into the lipid bilayer. In contrast, the F-BAR domains of Syp1 and Bzz1 had little effect on membrane fluidity.

(B) Mutations in the N-terminal helix of Rvs161/167 BAR domain diminished its ability to decrease membrane fluidity, suggesting that the N-terminal helix of the Rvs161/167 inserts into the lipid bilayer.

(C) Membrane binding of Rvs161/167 BAR domain increased the lipid order at the interfacial region as detected by Laurdan generalized polarization. Bzz1 and Syp1 F-BAR domains, on the other hand, displayed only small effects on lipid order.

(D) Mutations at the N-terminal helix of Rvs161/167 BAR domain diminished its ability to increase lipid order, suggesting that membrane insertion of the N-terminal helix plays an important role in this process.

(E and F) The effects of Rvs161/167 BAR domain on membrane fluidity and lipid order are dependent on lipid composition of the membrane. The BAR domain of Rvs161/167 affect the membrane fluidity and lipid order only in the presence of PI(4,5)P_2, suggesting that the N-terminal helix inserts into lipid bilayer only in phosphoinositide-containing membranes. The lipid composition in panels A-D was POPC:POPE:POPS: PI(4,5)P_2 = 50:20:20:10. DPH and Laurdan were incorporated into vesicles with 1:500, and 1:1000 ratios, respectively. The lipid concentration was 40 μM.

Cell PRESS
Open ACCESS

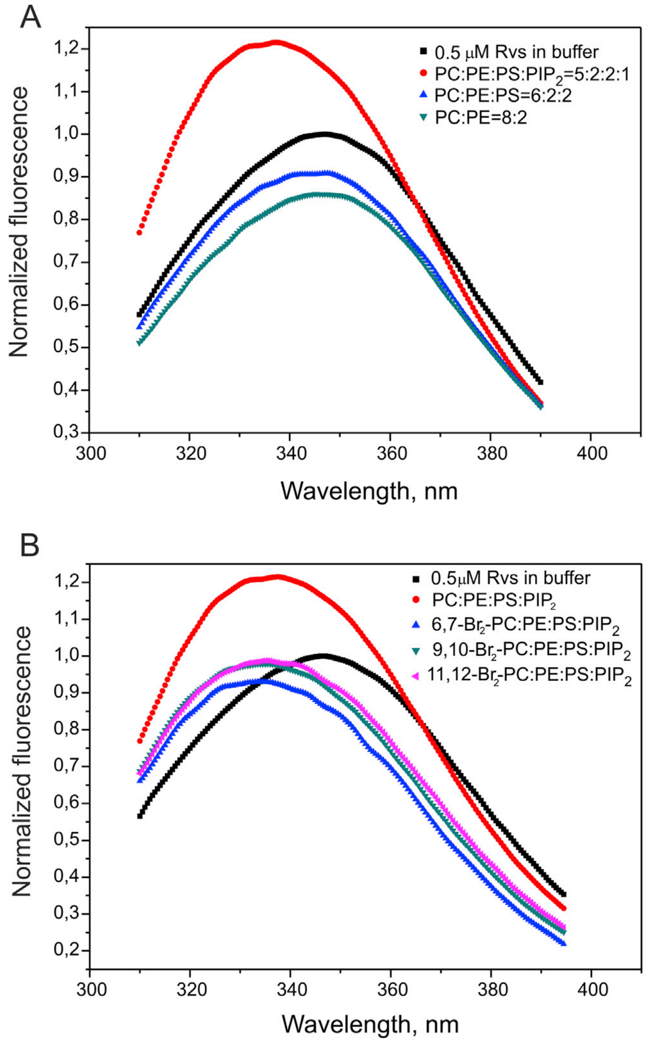

Figure S4. The N-Terminal α Helix of Rvs 161/167 BAR Domain Penetrates into the Acyl Chain Region of Lipid Bilayer, Related to Results

(A) The fluorescence intensity of Trp3 of Rvs 161 BAR domain increased and the emission spectra was blue-shifted in PI(4,5)P_2-containing membranes, suggesting that Trp3 is exposed to a more hydrophobic environment caused by the membrane insertion of the N-terminal helix. The concentrations of protein and lipid were 0.5 μM and 100 μM, respectively.

(B) To determine whether the N-terminal α-helix of Rvs161 indeed penetrates into the acyl chain region of the bilayer, we used phospholipids brominated along the acyl chain at different positions and measured collisional quenching of the Rvs161 Trp3 by bromides in the hydrophobic core region of the lipid bilayer. This experiment revealed that Trp3 fluorescence was quenched by bromides along the lipid acyl chain, providing evidence that the N-terminal α-helix of the Rvs161 inserts into the acyl chain region of the bilayer as recently proposed (Youn et al., 2010). Calculations via the parallax method suggest that Trp3 locates at a depth of ~10.3 Å from the center of the bilayer. The lipid composition was brominated-PC:POPE:POPS:PI(4,5)P_2 = 50:20:20:10. The concentrations of protein and lipid were 0.5 μM and 100 μM, respectively.

Cell PRESS
Open ACCESS

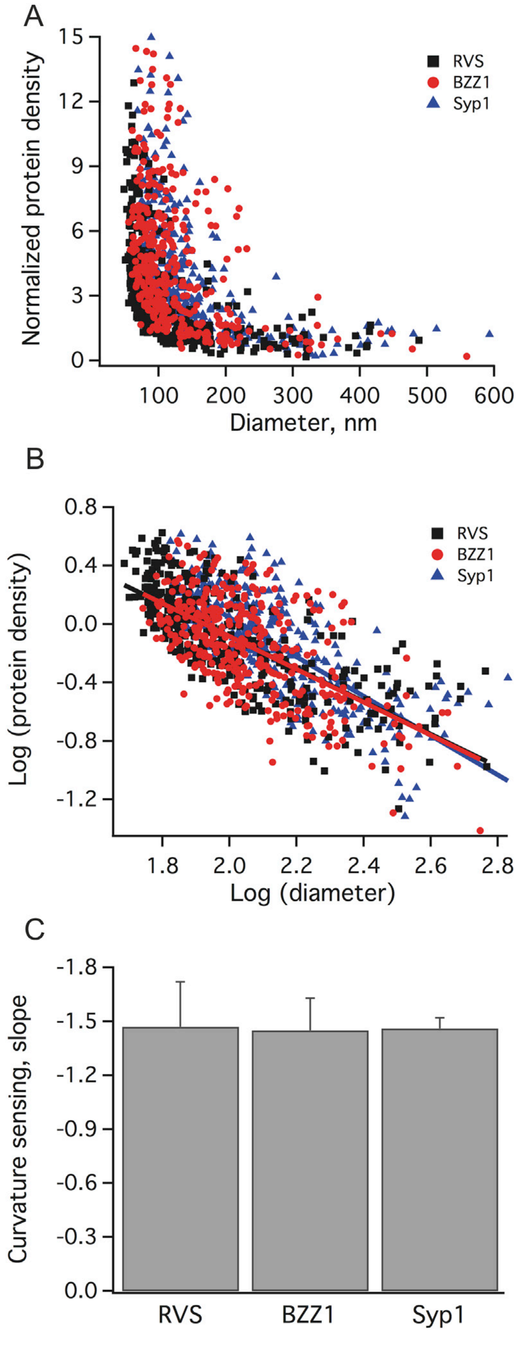

Figure S5. Membrane Curvature-Sensing Properties of Syp1, Bzz1, and Rvs161/167 BAR/F-BAR Domains, Related to Results

(A) Representative protein density versus liposome diameter for BAR domain of RVS 161/167, and F-BAR domains of Bzz1 and Syp1 measured at concentration of 1 μM. The density of the proteins, which is normalized to 1 for diameters > 300 nm, increases sharply for the highly curved liposomes (<100 nm).

(B) Plotting the protein density on a log-log scale allows the quantitative measurement of the power-law exponents and thus the sensing ability (Hatzakis et al., 2009). All tested proteins show an r^{α} behavior.

(C) There is no difference in the α values among the tested proteins. The error bars represent the SD of different experiments (n = 7). The lipid composition was POPC:POPE:POPS:PI(4,5)P_2:DOPE-biotin:DiO-C18 = 55:19:20:5:0.5:0.5.

Cell PRESS
Open ACCESS

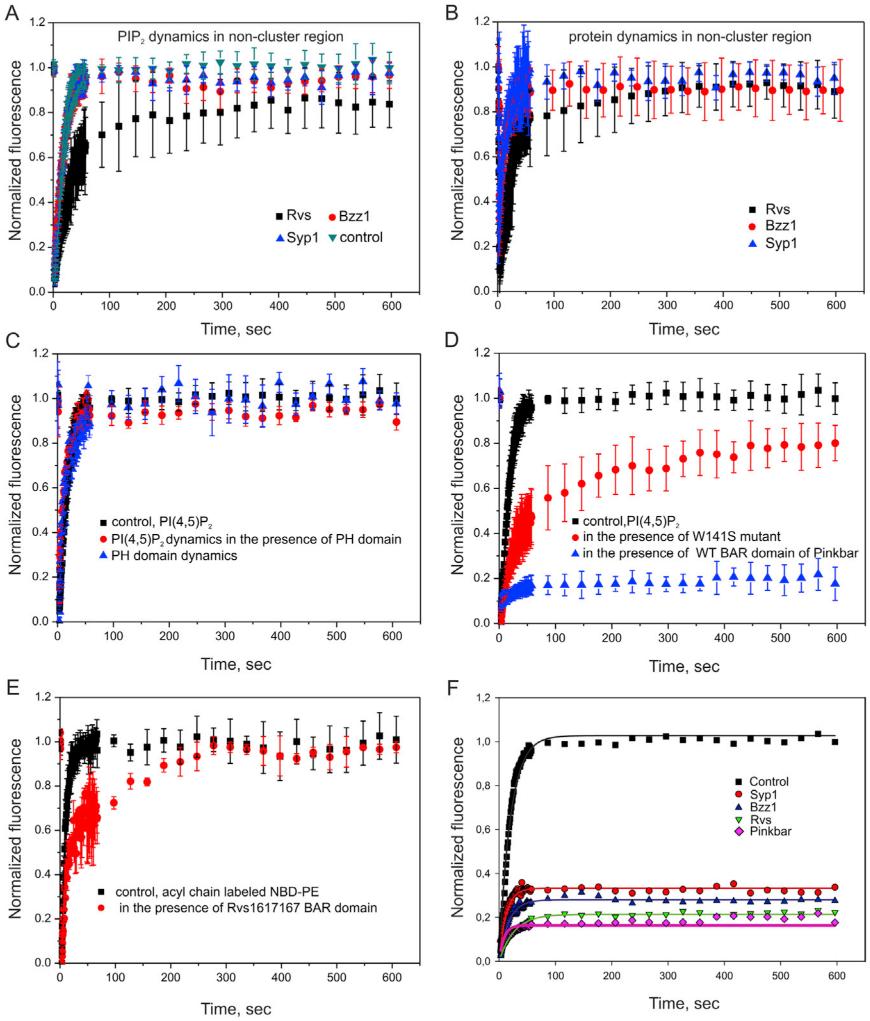

G

	$PI(4,5)P_2$		Protein	
	Fmobile, %	half time $t_{1/2}$, sec	Fmobile, %	half time $t_{1/2}$, sec
Control	100±2.08	13.4±2.38	-	-
Syp1	33.5±19.08	9.54±1.93	39.5±12.9	4.05±1.49
Bzz1	29.6±14.2	9.03±1.8	42.9±21.8	5.15±0.8
Rvs161/167	22.07±11.3	21.5±12.5	31.5±16.9	15.2±9.36
Pinkbar	16.2±8.5	8.7±2.42	11.2±6.48	3.65±2.13

Figure S6. Dynamics of the Endocytic BAR/F-BAR Domains and $PI(4,5)P_2$ in the Nonclustered and Clustered Regions of GUVs, Related to Results

(A and B) The dynamics of $PI(4,5)P_2$ (A) and the BAR/F-BAR domains of Rvs 161/167, Syp1 and Bzz1 (B) in non-clustered planar regions of GUVs were measured by FRAP. In contrast to extremely slow dynamics of protein and lipids in the membrane clusters/tubules (see Figure 3), these BAR/F-BAR domains displayed rapid dynamics in the non-clustered regions of GUVs, and the lateral diffusion of $PI(4,5)P_2$ was only slightly diminished compared to control vesicles without proteins.

(C) A FRAP assay on a mCherry-fusion of PLCδ PH domain revealed that unlike BAR domains, the PH domain displays rapid dynamics on GUVs and does not display detectable effects on the lateral diffusion of $PI(4,5)P_2$.

(D) A Pinkbar BAR domain mutant (W141S), which binds phosphoinositide-rich membranes with similar affinity to the wild-type BAR domain of Pinkbar but is less efficient in forming oligomers, is significantly less efficient in inhibiting the lateral diffusion of $PI(4,5)P_2$ compared to the wild-type domain.

(E) Dynamics of the acyl-chain labeled PE displayed only approximately 5-fold decrease in lateral diffusion in BAR domain clusters compared to control vesicles, and unlike head group labeled PE it reached full fluorescence recovery during the 10 min monitoring period.

(F) Data from the averaged fluorescence recovery curves (from 5-9 independent experiments, see Figure 3D) in the clustered regions of membranes were fitted to a single exponential equation (see ‘Materials and methods’).

(G) The mobile fractions and $t_{1/2}$ values ($\pm$standard deviation) were calculated from the fitted data for the individual recovery curves. The lipid composition was POPC:POPE:POPS:$PI(4,5)P_2$:TopFluor-$PI(4,5)P_2$ = 50:20:20:9:1. The protein concentrations were 1μM.
